# Association between viral infection and bronchopulmonary dysplasia in preterm infants: a systematic review and meta-analysis

**DOI:** 10.1007/s00431-024-05565-9

**Published:** 2024-04-18

**Authors:** Xin Guo, Defei Ma, Rui Li, Ruolin Zhang, Yanping Guo, Zhangbin Yu, Cheng Chen

**Affiliations:** 1 Department of Neonatology, Longgang District Maternity & Child Healthcare Hospital of Shenzhen City (Longgang Maternity and Child Institute of Shantou University Medical College), Shenzhen, 518172 Guangdong China; 2 Department of Neonatology, Nanshan Maternity & Child Healthcare Hospital, Shenzhen, 518067 Guangdong China; 3 Department of Pediatrics, Peking University Shenzhen Hospital, Shenzhen, 518036 Guangdong China; 4 Department of Neonatology, Shenzhen People’s Hospital, The Second Clinical Medical College of Jinan University, First Affiliated Hospital of Southern University of Science and Technology, Shenzhen, 518000 Guangdong China

**Keywords:** Bronchopulmonary dysplasia, Viruses, Preterm infants, Viral infections, Cytomegalovirus

## Abstract

Bronchopulmonary dysplasia (BPD) is the most common serious complication of very preterm infants (VPI) or very low birth weight (VLBW) infants. Studies implicate viral infections in etiopathogenesis. The aim of this study was to summarize the relationship between viral infections and BPD through a systematic review and meta-analysis. We searched PubMed, Embase, the Web of Science Core Collection, and the Cochrane Database on December 19, 2023. We included observational studies that examined the association between viral infections and BPD in preterm infants. We extracted data on study methods, participant characteristics, exposure assessment, and outcome measures. We assessed study risk of bias using the Newcastle-Ottawa Scale (NOS). We included 17 and 15 studies in the qualitative review and meta-analysis, respectively. The meta-analysis showed a significant association between viral infection and BPD diagnosed at 36 weeks postmenstrual age (odds ratio (OR): 2.42, 95% confidence interval: 1.89–3.09, 13 studies, very low certainty of evidence). In a subgroup analysis of specific viruses, cytomegalovirus (CMV) proved to be significantly associated with BPD diagnosed at 36 weeks postmenstrual age (OR: 2.34, 95% confidence interval: 1.80–3.05, 11 studies). We did not find an association between viral infection and BPD diagnosed on the 28th day of life, probably due to the small sample size of the included prospective studies.

*  Conclusion*: Viral infections, especially CMV, are associated with an increased risk of BPD in preterm infants. Methodologically reliable prospective studies with large samples are needed to validate our conclusions, and high-quality randomized controlled studies are needed to explore the effect of prevention or treatment of viral infections on the incidence of BPD.

**Table:** 

**What is Known:** *• Studies have attempted to identify viral infections and bronchopulmonary dysplasia in preterm infants; however, results have been inconsistent.*	
**What is New:** *• Systematic demonstration that viral infections, particularly cytomegalovirus, are positively associated with bronchopulmonary dysplasia diagnosed in preterm infants at the 36th week of postmenstrual age.* *• The importance of screening for viral infections in preterm infants, especially cytomegalovirus. More high-quality studies should be produced in the future to investigate the causal relationship between viral infections and bronchopulmonary dysplasia.*

**Supplementary Information:**

The online version contains supplementary material available at 10.1007/s00431-024-05565-9.

## Introduction

 Bronchopulmonary dysplasia (BPD) is the most common serious complication of very preterm infants (VPI) or very low birth weight (VLBW) infants, affecting up to 45% of infants born at less than 29 weeks gestational age (GA) [1]. There is a strong correlation between the severity of BPD and mortality, poor respiratory prognosis, persistent pulmonary hypertension, and neurodevelopmental impairment (NDI) in preterm infants [2–5]. A Canadian investigation found that BPD and its associated complications result in an extreme cost burden early in life and a lifelong negative impact on quality of life for preterm infants with a GA ≤ 28 weeks [6]. The pathogenesis of BPD is widely recognized as multifactorial. Sepsis-associated systemic inflammation and microbial infections may play a propulsive role in the pathogenesis of BPD [7]. A large Brazilian study found an association between the need for prolonged mechanical ventilation, treated patent ductus arteriosus (PDA), and delayed sepsis with an increased risk of BPD [8]. Bacteria and Mycoplasma solani are two infectious agents that have been widely mentioned in BPD-related studies, while relatively few studies have been conducted on viruses [9–11]. Although bacterial infections and Mycoplasma solani infections are more common than viral infections in preterm infants, the role of viral infections in BPD cannot be ignored.

Since there is a clear concept of time for the diagnosis of BPD, the hypothesis of viral infection as an etiology of BPD is based on observations in a cohort describing outcomes of preterm infants in the neonatal intensive care unit (NICU). Two studies found an association between acute lung injury due to seasonally prevalent mixed respiratory viral infections and the development of BPD [12, 13]. Couroucli et al. also found an association between adenovirus infection and the development of BPD [14]. Certainly, cytomegalovirus (CMV) should be the most discussed viral infection in BPD research. Large multicenter studies in the USA and Australia have concluded that CMV is a risk factor for BPD [15, 16].

However, there are some studies that take the opposite view. Prösch et al. from Germany concluded that adenovirus infection, CMV infection, and BPD are not related [17]. Large multicenter studies, also from the USA and Australia, also concluded that CMV infection had no significant effect on BPD [18, 19]. Most relevant studies are limited by small sample sizes, which prevent us from drawing definitive conclusions [20]. Therefore, we aimed to address the above paradox by summarizing studies assessing the relationship between viral infections and BPD in preterm infants through a systematic review and meta-analysis. The primary objective of our review was to determine whether viral infection is associated with the development of BPD in preterm infants compared with those without viral infection. A secondary objective was to determine the association between infection with different types of viruses and BPD in preterm infants.

## Methods

The system review adhered to the Preferred Reporting Items for Systematic Reviews and Meta-Analyses (PRISMA) statement [21] and the Meta-analysis Of Observational Studies in Epidemiology (MOOSE) guidelines [22]. A comprehensive review protocol, which includes objectives, eligibility criteria, information sources, and search strategies, has been registered with the International Prospective Register of Systematic Reviews (registration number: CRD42023493157). The systematic evaluation was a secondary analysis of the literature, and ethical review was considered unnecessary.

### Inclusion and exclusion criteria

We included cohort and case-control studies that examined the association of viral infections with BPD in preterm infants. We excluded reviews, case reports, case series, letters and editorials, and randomized controlled studies. We reviewed the reference lists of the included studies to identify additional studies. The participants, exposures and risk factors, comparison groups, and outcomes (PECO) of our studies are listed below.

### Participants

We included preterm infants born with a gestational age of less than 37 weeks who were admitted to the neonatal ward. We excluded all infants known to have congenital malformations.

### Exposure and risk factor

Preterm infants with viral infections detected in the respiratory tract or blood at or before the time of BPD diagnosis. We defined viral infection as the isolation or molecular detection of DNA or RNA of a causative virus known to affect the lungs from a laboratory specimen of the infant.

### Comparator

All preterm infants diagnosed with BPD who were not infected with the virus on or before the diagnosis of BPD.

### Outcome

Our primary outcome indicator was based on preterm infants still requiring oxygen at 28th day of age after birth (guidelines issued by the National Heart, Lung, and Blood Institute (NHLBI) and the National Institute of Child Health and Human Development (NICHD) in 2001) or at 36 weeks postmenstrual age (guidelines published by NICHD in 2018 or diagnostic criteria proposed by Jensen et al.) [23–25]. Literature with unclear diagnostic criteria or with chest imaging alone as a diagnostic criterion were reviewed qualitatively only and were not included in the meta-analysis [26, 27].

### Information sources

Our search date was 19 December 2023, and the search strategy had no language or publication date restrictions. We searched the following databases: PubMed (data inception to 19 December 2023), Embase database (data inception date to 19 December 2023), Web of Science Core Collection (data inception to 19 December 2023), and the Cochrane Library (data inception to 19 December 2023). We manually checked references in the included studies.

### Search strategy

The search strategies for the above four databases were developed by the reviewer (X.G.) and reviewed by other reviewers. The full search strategies are detailed in Appendix 1 of the Supplemental Information.

### Selection process

Two reviewers (X.G. and D.M.) independently reviewed the abstracts and screened and included studies according to inclusion and exclusion criteria. Conflicts were resolved by a third author (Z.Y.). We used a free online tool, DeepL (https://www.deepl.com/translator#zh/en/), to assess potentially eligible studies in non-English languages.

### Data collection process

Two reviewers (X.G. and D.M.) independently collected data by manually reviewing the included articles using a data extraction form. Z.Y. reviewed the data collected by the two reviewers to rule out any human error. For the studies included in this review where reports are ambiguous or data is incomplete, we will contact the authors to attempt to obtain full reports or data. A list of all data entries collected is detailed in Appendix 2 of the Supplemental Information.

### Study risk of bias assessment

We used the Newcastle-Ottawa scale to assess the risk of bias for the included cohort and case-control studies. Two reviewers (X.G. and D.M.) independently scored the studies they each screened during the data extraction process. The domains scored included selection, comparability, exposure, or outcome, with a maximum score of 4, 2, and 3 for each domain, respectively. The maximum possible total score for each study was 9.

### Data analysis and effect measures

We conducted a meta-analysis of the literature on those who reported data on viral infections in BPD and control groups. To calculate the odds ratio (OR) of the association between viral infection and BPD in different studies, we used the inverse variance method. Forest plots were used to visualize the results of the meta-analysis. Heterogeneity between studies was assessed using Cochran’s *Q* test and *I²* statistic. If the heterogeneity statistic *I²* was greater than or equal to 50% or the *P* value of the Cochran’s *Q* test statistic was less than 0.05, indicating significant heterogeneity between studies. Given that the included studies are primarily either prospective or retrospective observational studies, significant clinical or methodological differences may exist between them. Therefore, a random effects model was chosen for the meta-analysis. When the number of studies included is ten or more, publication bias is assessed by examining the asymmetry of funnel plots and conducting Egger’s and Begg’s tests. Meta-analyses, Egger’s and Begg’s tests, forest plots, and funnel plots were performed with R version 4.2.1 (R Foundation for Statistical Computing, Vienna, Austria).

## Results

### Characteristics of the included studies

**Fig. 1:**
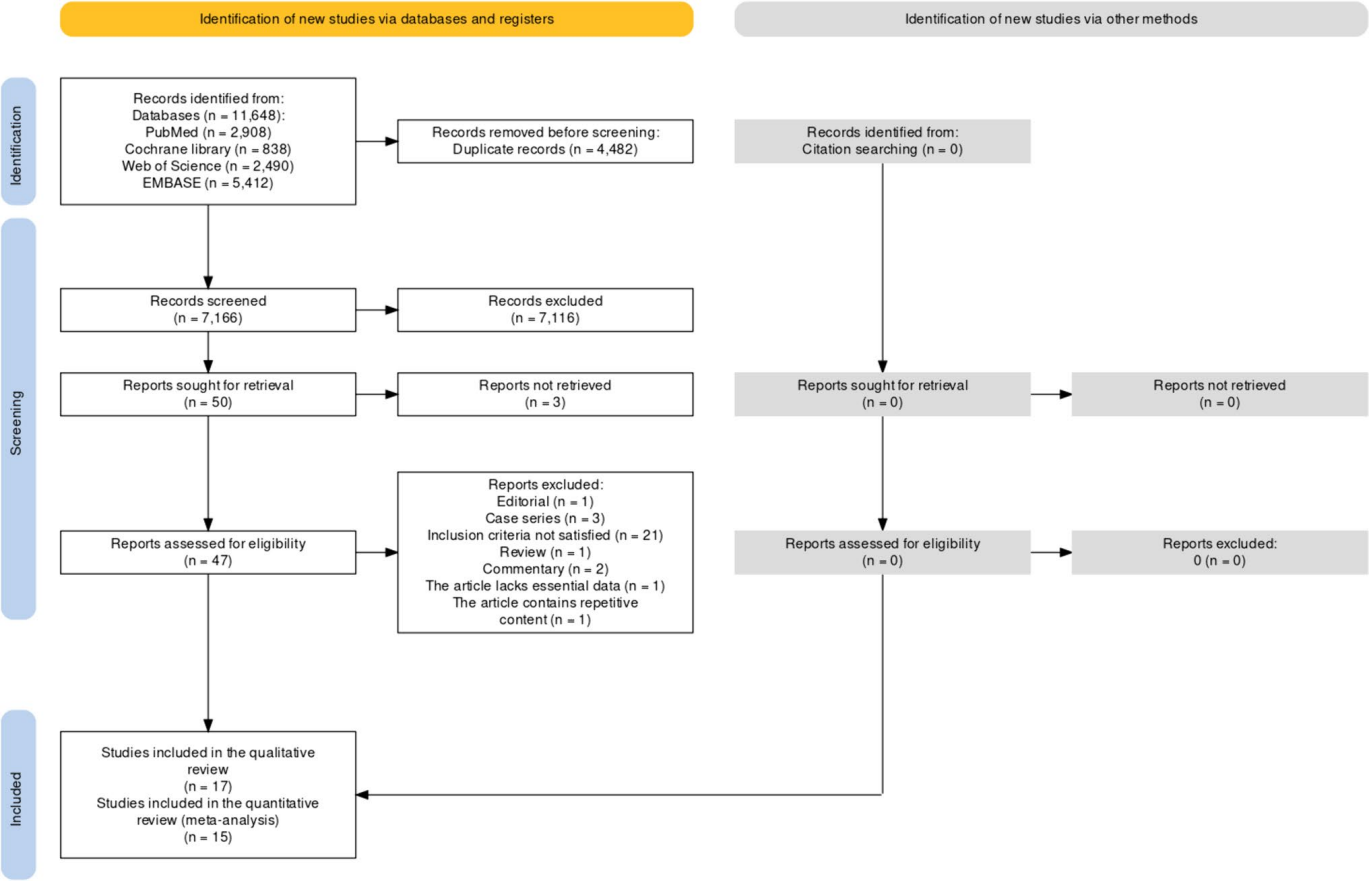
PRISMA flowchart shows the systematic search of the literature

In total, we screened 7166 potentially relevant study abstracts, of which 50 were selected for full-text search, and 17 studies were ultimately included in the systematic review. The PRISMA flowchart of the search and screening process is shown in Fig. 1. The included studies and their baseline characteristics are summarized in Table 1 [12–18, 20, 26–34]. Excluded studies and the reasons for their exclusion are shown in Supplemental Table 1. Fifteen studies were published in 2001 and later, of which 64.7% were published within the last 12 years (Supplemental Fig. 1A). The included studies were conducted primarily in five countries around the world. The USA and Germany were the main study sites, and 76.5% of the studies were conducted in these locations (Supplementary Fig. 1A, B). All studies were cohort studies (47.1% prospective, 52.9% retrospective). The included studies evaluated 7734 preterm infants to explore the association between different viruses and BPD. CMV was the most studied virus, comprising 76.5% of the studies (Supplementary Fig. 1A).

**Table 1:** Baseline characteristics of the included studies

**Study no.**	**Study ID**	**Study period**	**Study design**	**Location**	**Participants**	**GA (weeks), mean (SD), median (IQR) or range**	**BW (grams), mean (SD) or range**	**Exposure ascertainment**	**Definition of outcome**	**Sample size**
1	Bennett et al. (2012) [12]	2009	Prospective cohort	USA	Infants with GA ≤ 33 weeks 0 days	28.7 (3.0)	1322 (770)	Infants’ respiratory secretions were repeatedly tested before discharge using multiplex PCR containing 17 different respiratory viruses or subtypes	Infant required supplemental oxygen at 36 weeks post-conceptional age	50
2	Bimboese et al. (2022) [28]	August 15, 2001–December 16, 2003	Prospective cohort	Australia	Preterm infants born to mothers who tested positive for CMV IgG	27.1 (1.9)	914 (197)	Infants were considered to have postnatal CMV if they had at least one positive urinary CMV PCR within the first 14 weeks of life	Fraction of inspired oxygen > 0.21 at 28 day of life or 36 weeks of GA	58
3	Capretti et al. (2009) [29]	December 2003–August 2006	Prospective cohort	Italy	All newborns of CMV IgG-positive mothers with a birth weight (BW) < 1500 g and GA < 32 weeks who were breastfed	28.7 (2.1)	1104 (282)	Isolation of CMV from breast milk and isolation of CMV from infant urine with positive urine CMV qualitative PCR and matching of CMV gN genotype in infant urine to that in breast milk	Oxygen requirement at the age of 36 weeks post-conceptional age	62
4	Couroucli et al. (2000) [14]	11 month (1997–1998)	Prospective cohort	USA	All infants electing intubation at birth with a GA < 30 weeks	< 30	NA	The PCR test for adenovirus in a tracheal aspirate sample within 7 days after birth was positive	Oxygen dependency at 28 day of life or 36 weeks postconceptional age	76
5	Hernandez-Alvarado et al. (2021) [20]	October, 2014 - July, 2019	Retrospective cohort	USA	VLBW preterm infants (BW < 1500 g) admitted to the NICU and whose mothers plan to breastfeed them	28.1 (26.0–29.8)	1020 (760–1210)	Infants with CMV DNAemia by PCR or positive CMV IgM assay by ELISA	Oxygen therapy at 36 weeks corrected gestational age	75
6	Humberg et al. (2018) [30]	1st of January 2009–31st of December 2014	Retrospective cohort	Germany	Those VLBW infants from whom umbilical cord tissue was collected	28.6 (2.7)	1046 (304)	CMV-specific PCR in umbilical tissue samples obtained from VLBW infants	Needing supplemental oxygen at 36 weeks of post menstrual age	3330
7	Inagaki et al. (2019) [26]	January 1–December 31 in 2003, 2006, 2009, and 2012	Retrospective cohort	USA	Preterm infants with GA ≤ 32 weeks from public databases	≤ 32	<2500	Searching for International Classification of Diseases, Ninth Revision, Clinical Modification (ICD9-CM) code 771.1 (congenital cytomegalovirus infection)	Searching for International Classification of Diseases, Ninth Revision, Clinical Modification (ICD9-CM) code 770.7 (bronchopulmonary dysplasia)	687
8	Kelly et al. (2015) [34]	1997–2012	Retrospective cohort	USA	VLBW infants hospitalized on day of life 21 in NICUs	<32	<1500	CMV detected by culture or PCR in blood, urine, cerebrospinal fluid, or respiratory secretions of infants < 36 weeks GA on or after 21 days of age	VLBW infants receiving continuous respiratory support between 36 0/7 weeks and 36 6/7 weeks postmenstrual age	606
9	Meier et al. (2005)^a^ [31]	May 1999–May 2003	Prospective cohort	Germany	Breastfeeding mothers and their preterm infants	24–33	380–2010	Detection of CMV IgG and IgM antibodies in serum using enzyme-linked immunosorbent assay (ELISA) and detection of CMV DNA in breast milk, tracheal aspirates, pharyngeal aspirates, and urine using PCR	Oxygen requirement at 36 weeks of postconceptional age or oxygen requirement of 25% on 28th day of life	89
10	Mukhopadhyay et al. (2016) [32]	January 1, 1999–December 31, 2013	Retrospective cohort	USA	All live-born VLBW infants who remained in the NICU at 21 days of age	27.4 (2.2)	923 (200)	CMV has been detected in urine, blood, cerebrospinal fluid (CSF), or tracheal secretions by shell vial assay and viral culture	Requirement for supplemental oxygen and/or respiratory support at 36 weeks’ gestation	1080
11	Neuberger et al. (2006) [33]	June 1, 1995–December 31, 2003	Retrospective cohort	Germany	Infants with a BW < 1000 g or gestation < 30 weeks and whose mothers are seropositive for CMV. Prior to June 1998, infants with a GA of 30 to 31 weeks or a BW of 1000 to 1500 g	24.1–31.7	495–1670	CMV PCR-positive urine sample	Oxygen requirement at 36 weeks gestation	80
12	Prösch et al. (2002)^a^ [17]	May 1999–October 2000	Prospective cohort	Germany	Preterm infants	24–32	380–1500	Detection of human adenovirus in tracheal aspirate, pharyngeal aspirate, and urine by PCR and detection of human adenovirus in tracheal aspirate and pharyngeal aspirate by virus culture	Oxygen dependency at day 28 of life	66
13	Sánchez Garciá et al. (2020) [13]	April 2016–March 2018	Prospective cohort	Spain	Infants with GA < 32 weeks and admitted to the NICU within 72 h of birth	28.5 (2.1)	1100 (320)	Detection of respiratory viral nucleic acids in nasopharyngeal aspirates of infants using four different real-time multiplex PCR techniques	Treatment with oxygen > 21% for at least 28 days and severity was categorized at 36 weeks of postmenstrual age or discharge	147
14	Sawyer et al. (1987) [27]	April 1979–April 1984	Prospective cohort	USA	All premature infants	25–33	570–1960	Urine cultures for CMV excretion	Serial chest roentgenograms demonstrating typical and chronic parenchymal abnormalities	64
15	Tapawan et al. (2023) [15]	January 2007–December 2019	Retrospective cohort	Australia	Preterm infants with GA < 32 weeks	26.1 (2.1)	831 (266)	Positive CMV PCR test result from either urine, blood, or respiratory secretions after 21 days of life	Continued need for any form of respiratory support (supplemental oxygen and/or assisted ventilation) beyond 36 weeks postmenstrual age	344
16	Turner et al. (2014) [18]	January 1993–December 2008	Retrospective cohort	USA	All congenital or transported preterm infants with a BW < 1500 g	27.7 (2.9)	983 (1530)	Congenital CMV infection detected by rapid urine or saliva culture within 2 weeks. Postpartum CMV infection detected by rapid urine or saliva culture after 2 weeks	Oxygen requirement at 36 weeks’ postmenstrual age	374
17	Weimer et al. (2020) [16]	January 1, 2002–December 31, 2016	Retrospective cohort	USA	Infants with BW < 1500 g who were still hospitalized on the 21st day of life and had hearing screening results after 34 weeks of postmenstrual age	≤ 32	<1500	Diagnosis of CMV infection, congenital CMV, acquired CMV infection, or detection of CMV in blood, urine, cerebrospinal fluid, or respiratory secretions by viral culture or PCR on or after postnatal day 21	Infants with GA < 32 weeks received continuous respiratory support between postmenstrual age 36 weeks 0 days and 36 weeks 6 days. Infants with GA of at least 32 weeks receive supplemental oxygen or respiratory support from postnatal days 28 to 34	546

^a^The data related to CMV infection in the Meier et al. [31] article incorporates the data related to CMV infection in the Prösch et al. [17] article

The population included in the studies was primarily preterm or low birth weight infants. Among the 17 incorporated studies, preterm infants in six investigations demonstrated gestational ages below 32 weeks [13–15, 29, 33, 34], whereas those in eight studies exhibited birth weights under 1500 g [16, 18, 20, 29, 30, 32–34]. One study did not report the birth weight of the infants studied [14]. Five studies reported outcomes for BPD diagnosed at postnatal age 28 days [13, 14, 17, 28, 31], and thirteen studies reported outcomes for BPD diagnosed at 36 weeks of postmenstrual age [12, 14–16, 18, 20, 28–34]. One study extracted data from a database using diagnostic codes only and did not account for diagnostic criteria for BPD [26]. One study diagnosed BPD relying solely on the results of chest imaging [27]. When selecting samples to confirm viral infection, eleven studies chose urine samples [15–18, 27–29, 31–34], nine used respiratory secretion samples [12–17, 31, 32, 34], six used blood samples [15, 16, 20, 31, 32, 34], three chose cerebrospinal fluid samples [16, 32, 34], and one took umbilical cord tissue samples [30]. In choosing the means of detecting viral infections, thirteen studies opted for nucleic acid testing by polymerase chain reaction (PCR) [12–17, 20, 28–31, 33, 34], seven took viral cultures [16–18, 27, 29, 32, 34], and two used immunological methods [20, 31]. One study performed genotyping in addition to nucleic acid testing [29].

The authors of six studies concluded that there was no association between viral infections and BPD [17, 18, 28, 29, 31, 33]. The authors of eleven studies concluded that viral infections were positively associated with BPD [12–16, 20, 26, 27, 30, 32, 34].

### Quality assessment

**Fig. 2:**
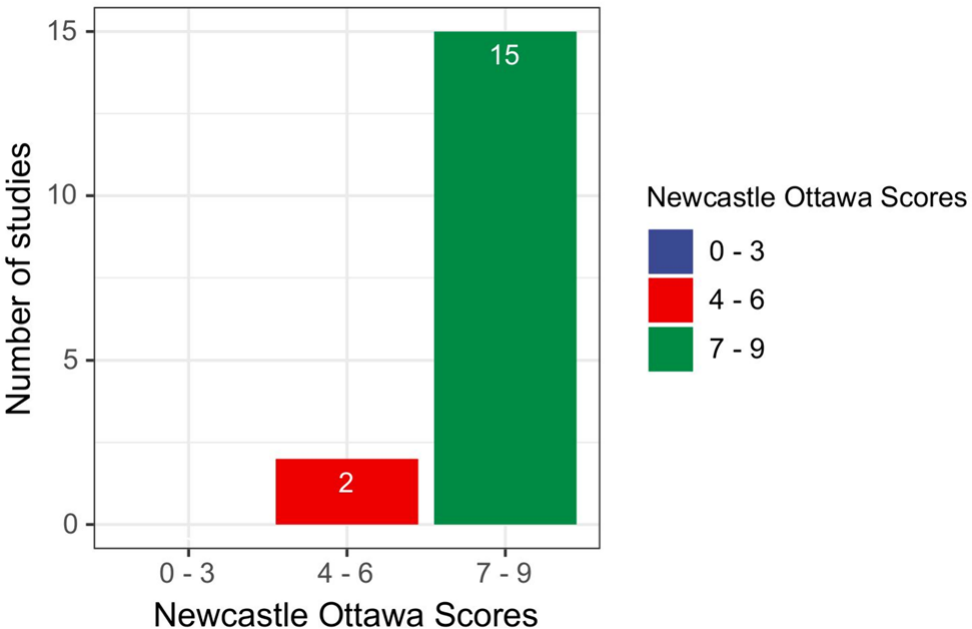
Risk of bias assessment. The column chart shows the Newcastle Ottawa Scores along the x-axis divided into 3 groups: high risk of bias (0–3), moderate risk of bias (4–6), and low risk of bias (7–9). The number of studies included in the review with those scores on the y-axis

The quality of each included study was assessed according to the Newcastle-Ottawa Scale (NOS), and the results are detailed in Supplemental Table 2. Based on past experience, literature rated 0 to 3 is considered high risk of bias, 4 to 6 is considered moderate risk of bias, and 7 to 9 is considered low risk of bias [35]. Agreement between the two independent reviewers (X.G. and D.M.) was 94%. Disagreement in one study was resolved by discussion and consensus. Fifteen studies received a score of 7 to 9 out of a possible 9 (Fig. 2).

Studies with high quality scores had clear criteria for selection of participants, comparability between groups, identification of exposure factors, and assessment of outcomes. The most common reason for lower scores on quality assessment was that studies did not control for important confounders such as birth gestational age and birth weight (two studies). We found asymmetry in our visual inspection of funnel plot (Supplemental Fig. 2). But the results of Begg’s test (BPD diagnosed at 36 weeks of postmenstrual age: *p*-value = 0.542) and Egger’s test (BPD diagnosed at 36 weeks of postmenstrual age: *p*-value = 0.556) demonstrated the absence of publication bias in meta-analyses.

### Meta-analysis

**Fig. 3:**
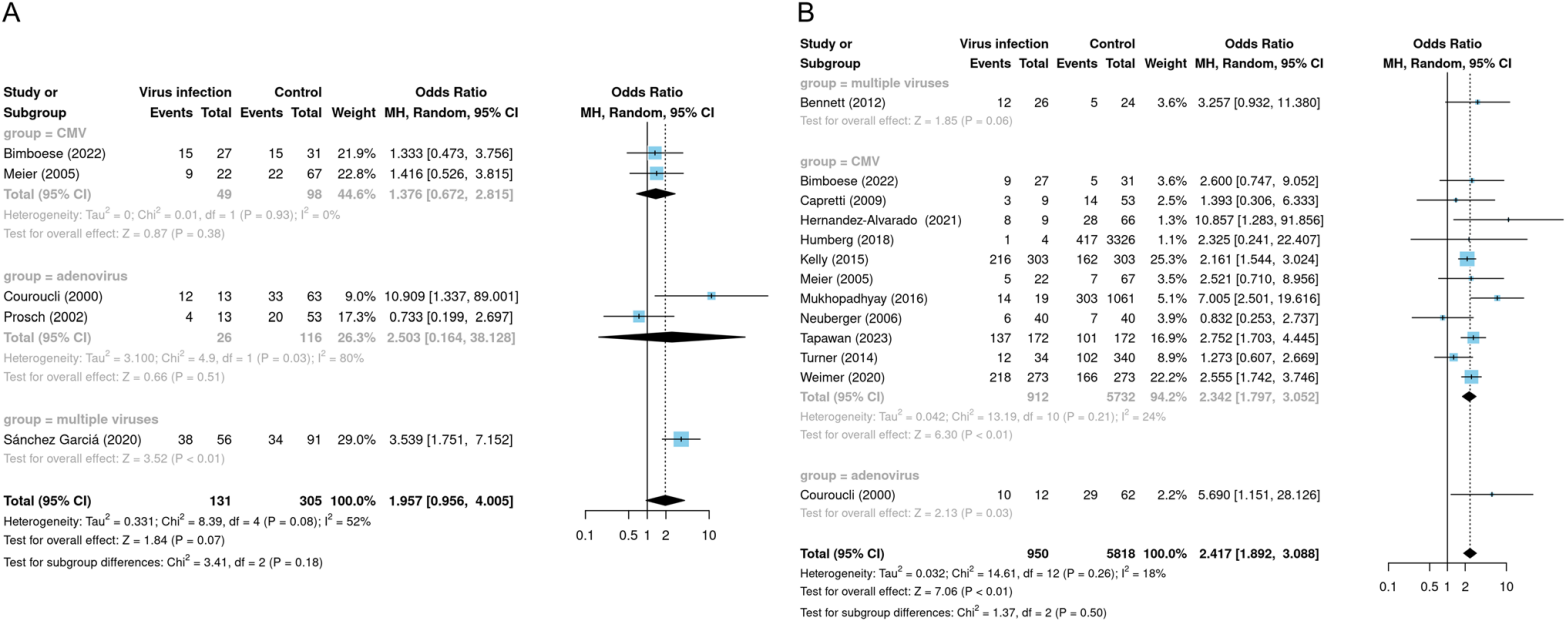
Meta-analyses and subgroup analyses. **A** Forest plot showing meta-analyses conducted with a diagnosis of BPD at 28th day of life as a positive outcome, with a random-effects model used to test the results of the meta-analyses. The forest plot also shows subgroup analyses by virus type. **B** Forest plot showing the meta-analysis performed with a diagnosis of BPD at 36 weeks postmenstrual age as a positive outcome, and the results of the meta-analysis were tested using a random-effects model. The forest plot also shows subgroup analyses by virus type

We included fifteen studies that provided data for the meta-analysis, three of which had two diagnostic criteria for BPD. Viral infection significantly increased the odds of a diagnosis of BPD in preterm infants at the 28th postnatal day of age (OR: 1.96, 95% confidence interval: 0.96–4.01), yet there was no significant difference in the analysis of the outcomes (*Z* = 1.84, *p*-value = 0.07), and there was significant heterogeneity between studies (*I*^*2*^ = 52%, *p*-value = 0.08) (Fig. 3A). Viral infection significantly increased the odds of diagnosis of BPD in preterm infants at 36 weeks of postmenstrual age (OR: 2.42, 95% confidence interval: 1.89–3.09), and there was a significant difference in the analysis of the outcomes (*Z* = 7.06, *p*-value < 0.01), and there was no significant heterogeneity between the studies (*I*^*2*^ = 18%, *p*-value = 0.26) (Fig. 3B).

### Subgroup analyses

**Fig. 4:**
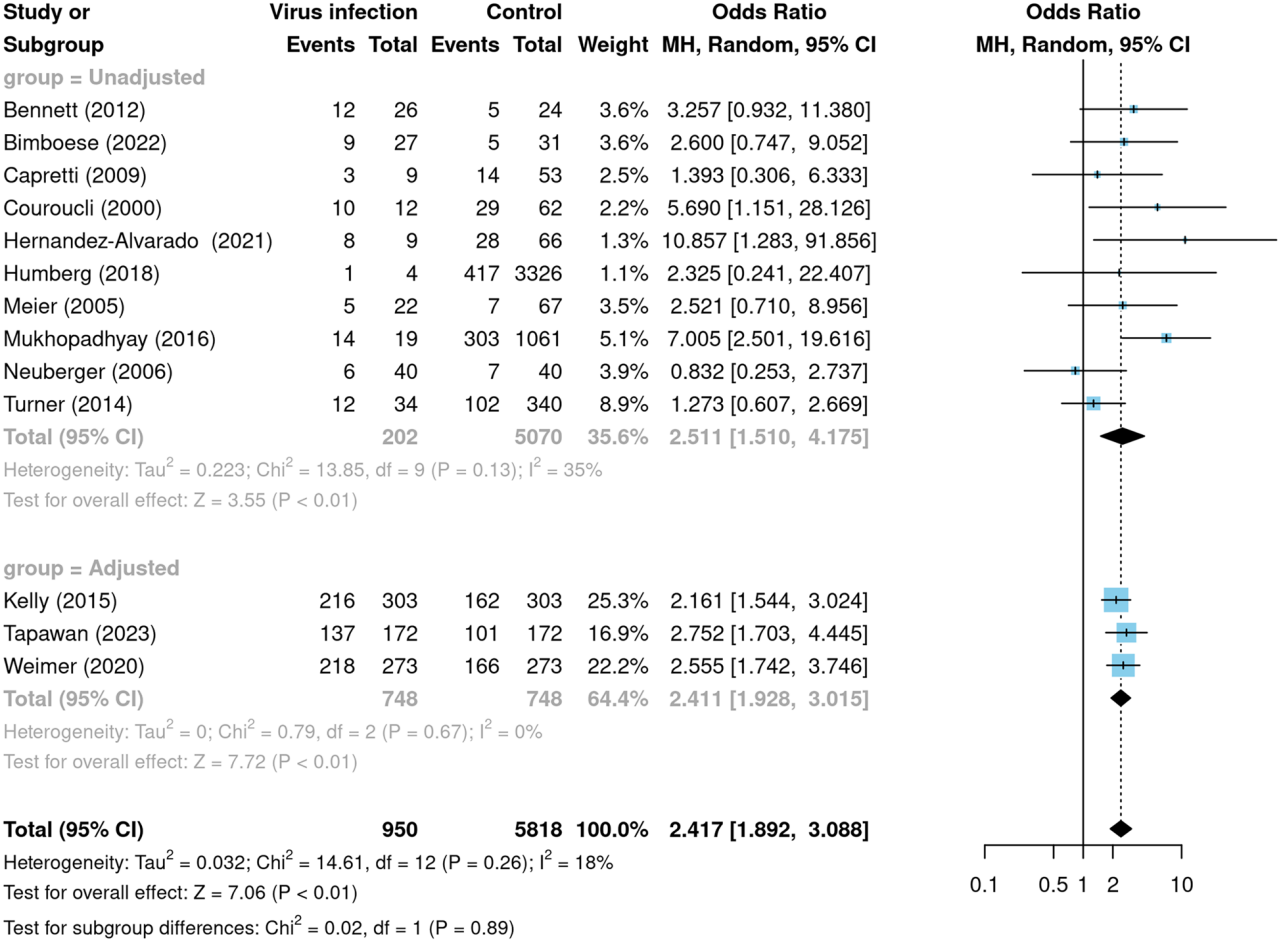
Subgroup analyses. The forest plot shows the subgroup analyses according to whether it was a proportion after adjusting for confounders, with the outcome being a diagnosis of BPD at 36 weeks after menstruation. The meta-analysis was examined using a random-effects model. Tapawan et al. [15] matched control infants by sex, birth gestational age, and birth weight. Kelly et al. [34] adjusted for confounding variables including GA, BW, small for gestational age status, race/ethnicity, discharge year, NICU site, use of antenatal corticosteroids, days of breast milk exposure from postnatal days 15 to 21, days of aminoglycoside (gentamicin sulfate, tobramycin, and amikacin sulfate) and loop diuretic (furosemide and bumetanide) exposure, NEC, grade 3 or 4 intraventricular hemorrhage, patent ductus arteriosus, sepsis occurring on or before postnatal day 21, vasopressor medications (amrinone lactate, dobutamine hydrochloride, dopamine hydrochloride, epinephrine, milrinone lactate, and norepinephrine bitartrate), type of respiratory support, and fraction of inspired oxygen on postnatal day 21. Confounding variables adjusted for by Weimer et al. [16] included GA, birth weight, small-for-gestational-age status, sex, race/ethnicity, discharge year, NICU site, days of breast milk exposure between postnatal day 15 and 21, number of days on which surfactant was received, necrotizing enterocolitis, grade III or IV intraventricular hemorrhage, PDA, and sepsis episode occurring on or before postnatal day 21, and number of vasopressor medications, type of respiratory support, and fraction of inspired oxygen assessed on postnatal day 21

We performed subgroup analyses according to different virus types. In studies where BPD was diagnosed at 28 days of age postnatally, subgroup analyses of CMV (OR: 1.38, 95% confidence interval: 0.67–2.82, *I²* = 0%) and adenovirus (OR: 2.50, 95% confidence interval: 0.16–38.13, *I²* = 80%) showed no significant association between viral infection and BPD (Fig. 3A). In the studies with BPD diagnosed at 36 weeks postmenstrual age, subgroup analysis of CMV (OR: 2.34, 95% confidence interval: 1.80–3.05, *I²* = 24%) showed a significant association between CMV infection and BPD, which was positively correlated (Fig. 3B). The portion of studies with a diagnosis of BPD at week 36 postmenstrual provided proportions based on propensity score matching after adjusting for confounders. We therefore conducted subgroup analyses according to the presence or absence of adjustment for confounders. The results showed that virus was consistently positively associated with BPD in both the adjusted for confounders subgroup (OR: 2.41, 95% confidence interval: 1.93–3.02, *I²* = 0%) and the unadjusted for confounders subgroup (OR: 2.51, 95% confidence interval: 1.51–4.18, *I²* = 35%), which were consistently positively correlated (Fig. 4). Heterogeneity was significantly less in the subgroup adjusted for confounders.

### Certainty of evidence

The results of our review are summarized in Table 2. We used the GRADEpro guideline development tool to categorize the certainty of the evidence as very low, low, moderate, or high based on study design, risk of bias, imprecision, inconsistency, indirectness, publication bias, large effect, plausible confounding, and dose-response gradient. In five studies that diagnosed BPD at 28 days of age, we found that the certainty of the evidence was very low. In thirteen studies of BPD diagnosed at 36 weeks postmenstrual age, we found that the certainty of the evidence was very low. The main reasons for the downgrading of the level of evidence were imprecise evidence due to the low total number of events included in the studies, non-randomized controlled cohort design, and publication bias. GRADEpro is a web-based tool for synthesizing and rating evidence that is widely used by medical organizations around the world, so we used it to derive this conclusion.

**Table 2:** Summary of data analysis

**Outcome**	**Number of studies (number of participants)**	**Risk of bias**	**Inconsistency**	**Indirectness**	**Imprecision**	**Other considerations**	**Statistical method**	**Effect estimate**	**Importance**	**Certainty of evidence (GRADE)**
BPD diagnosed at 28th day of postnatal age	5 (436)	Not serious	Not serious	Not serious	Serious^a^	Publication bias strongly suspected	OR (random, 95% CI)	1.957 (0.956–4.005)	Critical	Very low (+)
BPD diagnosed at 36 weeks of postmenstrual age	13 (6768)	Not serious	Not serious	Not serious	Not serious	Publication bias strongly suspected	OR (fixed, 95% CI)	2.417 (1.892–3.088)	Critical	Very low (+)

^a^Relatively few total number of events

## Discussion

Viral infections have been associated with BPD, but the conclusions of the available literature are not uniform. Evidence summarized in this systematic review and meta-analysis suggests that viral infection is significantly and positively associated with BPD diagnosed at 36 weeks of postmenstrual age and not significantly associated with BPD diagnosed on the 28th day of life. Based on subgroup analysis, CMV infection was significantly and positively associated with BPD diagnosed at 36 weeks of postmenstrual age. According to the GRADE assessment, the certainty of evidence for the association of viral infections with BPD was very low in studies with BPD diagnosed at both 28 days after menstruation and 36 weeks after menstruation.

Our meta-analysis covered 6983 infants, the largest sample to date to analyze the association of viral infections with BPD. This is the first review to synthesize data from all viruses hypothesized to be associated with BPD. By subgroup analysis, we further found that in the studies we included in the meta-analysis, only the positive association of CMV infection with BPD diagnosed at 36 weeks postmenstrual age was confirmed. The main reason for these conclusions is due to the small number and sample size of studies describing the association of adenoviruses and other viruses with BPD. A systematic review by Stark et al. found 10 studies supporting an increased risk of BPD in VLBW preterm infants infected with CMV, while 6 studies were against, which is consistent with our findings [36]. However, the systematic review by Stark et al. did not perform a meta-analysis of the relationship between CMV infection and the risk of developing BPD. Previous systematic reviews have shown that children with BPD are at higher risk for severe respiratory syncytial virus disease [37]. But there is a paucity of literature exploring the impact of common respiratory viruses such as adenovirus and respiratory syncytial virus on BPD in preterm infants. Our study identified four prospective cohort studies supporting that common respiratory viruses may have a potential association with the prevalence of BPD, but requires repeated validation and confirmation in large, multicenter prospective cohort studies [12–14, 17].

Infection, hyperoxia, and mechanical ventilation-induced lung inflammation are pivotal factors in the development of BPD. Studies on premature infants during the initial days of life have revealed that diminished levels and activity of surfactant protein D, a protein responsible for pathogen clearance and immune response regulation, correlate with adverse lung outcomes in BPD [38]. This protein plays a crucial role in mitigating lung tissue damage in children afflicted with BPD. The secretory phospholipase A2, which regulates inflammatory mediators and surface tension in the lungs, may be an important intermediary between viral infection and acute lung injury [39, 40]. In summary, the virus may exacerbate acute lung injury in BPD by regulating pulmonary inflammation, pulmonary compliance, and inhibiting viral clearance ability and immune response.

The definition of BPD has gone through a long process of adjustment. The study found that the 2018 NICHD diagnostic criteria and the 2019 Jensen diagnostic criteria better predicted the poor prognosis of severe respiratory disease, neurosensory disorders, and death in preterm infants compared to the 2001 NHLBI/NICHD diagnostic criteria [41–43]. Our study also found that viral infections predominantly showed significant differences in studies of BPD diagnosed at 36 weeks postmenstrual age. Therefore, we hypothesized that CMV infection is more strongly associated with more severe BPD and poorer prognosis.

There are three routes of transmission of CMV infection in preterm infants, including congenital CMV via intrauterine transmission, transmission via cervical or vaginal secretions, and postnatal CMV via breast milk or blood transfusion [15, 16]. Previous studies have defined congenital CMV infection using PCR or viral isolation (culture) for CMV DNA in body fluids (including saliva and/or urine) or blood obtained within 3 weeks of birth [44]. The current view is that the diagnosis of congenital CMV is made by detection of positive CMV DNA by PCR in samples collected within 21 days of birth, independent of viral culture and serum immunoantibody testing [45]. Congenital CMV infection was found to contribute to the development of BPD, suspected to be a direct or indirect effect of the virus on lung tissue [26, 30]. Not only that, preterm infants with congenital CMV infection are much more likely to develop progressive sensorineural hearing loss and poorer neurodevelopmental outcomes, which is a serious threat to the health of preterm infants [18]. Therefore, regular monitoring of fetuses whose mothers are positive and regular screening of high-risk babies after birth can contribute to early prevention and treatment [46]. There are many means of detecting postnatal CMV infection, including culture or PCR testing of biological samples such as urine, blood, and respiratory secretions [16]. However, current clinical practice and research would widely use a positive CMV DNA in a sample detected by PCR on and after the 21st day of life and a negative CMV DNA PCR within the first 21 days of life as the primary definition of postnatal CMV infection [15]. To demonstrate the risk of vertical transmission, it is advisable to test for CMV in breast milk at the same time. Most of the studies we included support a role for postpartum CMV infection as a driver of BPD, especially BPD diagnosed at 36 weeks postmenstrual age [15, 16, 20, 34]. Possible mechanisms by which postpartum CMV infection increases the risk of BPD include lung tissue damage, indirect immune response, and worsening of respiratory status exacerbating other BPD risk factors [34]. However, the sample sizes of the included prospective studies were generally too small. Retrospective cohort studies, although with slightly larger sample sizes, tested for CMV infection on the basis of clinical suspicion, leading to controls that may have been false-negative [19]. Therefore, our findings will have to be verified in large-sample, multicenter prospective studies.

CMV infections caused by blood transfusions have been greatly reduced because transfusions of CMV seronegative and leukopenic blood products have become common [47]. Therefore, it is now the mother’s breast milk that is the main source of postnatal CMV infection. Preterm infants infected with CMV postnatally, especially those with symptomatic infections, may develop long-term lung and neurodevelopmental morbidities [36]. Therefore, early dynamic monitoring of CMV DNA in breast milk is necessary for high-risk infants, such as VPI or VLBW infants and infants whose mothers are seropositive for CMV [20]. Among preterm and low birth weight infants, the prevalence of CMV infection was significantly higher in infants fed untreated breast milk than in infants fed frozen breast milk and mixed feeding [48]. However, frozen-thawed breastfeeding is not entirely effective in preventing postpartum cytomegalovirus infection [49]. In addition to freezing breastmilk, treating breastmilk with pasteurization is a means of interrupting the spread of pathogens [50]. Short-term pasturization significantly reduced the incidence of postnatal CMV infection through breast milk in the NICU [51]. In response to the fact that mothers of low birth weight babies may have insufficient breast milk of their own, human donor breast milk requiring pasteurization is widely used [52]. However, studies have found that pasteurized breast milk may negatively affect longitudinal growth and beneficial components in preterm infants [53, 54]. This also exemplifies the importance of performing the necessary postnatal CMV screening to rationally prevent breast milk transmission. Current clinical practice does not recommend routine screening of mothers for CMV infection during pregnancy, but serologic testing is recommended for symptomatic women or for women whose ultrasound demonstrates fetal intestinal fluid, brain abnormalities, or fetal size less than gestational age [55]. During breastfeeding, CMV is secreted into breast milk from the first week postpartum, with an initial low viral load that peaks at approximately 4–8 weeks, then declines and ends at 9–12 weeks postpartum [56]. Based on this characterization, for VPI and VLBW infants, it is recommended that CMV concentrations in breast milk be monitored at least every 1 to 2 weeks beginning in the first week of life. However, the frequency of screening of mothers and breast milk has not been clearly documented, and further high-quality studies are still needed to summarize the evidence. The guideline recommends early serologic screening of mothers for preterm infants with a GA of less than 32 weeks and short-term pasteurization of breastmilk in women who are seropositive for CMV [57]. This is because short-term pasteurization appears to minimize the impact on the beneficial components of breast milk while retaining the ability to inactivate the virus. Bedside, immediate breastmilk CMV testing may identify high-risk situations requiring pasteurization in real time. Other means include the use of anti-CMV immunoglobulin and/or leukocyte filtration treatment of breast milk [57]. Ongoing monitoring of CMV infection in at-risk populations while breastfeeding, such as weekly PCR testing of saliva, can help to identify infants who might benefit from “preventive” treatment at an early stage [58, 59].

Another very important but unresolved issue is the treatment of CMV infection. The use of valganciclovir was initiated in the neonatal period and even beyond to treat children with symptomatic or asymptomatic congenital CMV disease, improving hearing and developmental outcomes [60]. However, the literature examining the effect of anti-CMV viral therapy on BPD is sparse, with a lack of high-quality randomized controlled studies. Investigations by the German Neonatal Network have shown that infants with congenital CMV infection who require antiviral therapy are more likely to develop BPD and that antiviral therapy for CMV-negative infants leads to short-term adverse outcomes [30]. Ganciclovir treatment of postnatal CMV-infected VPI does not reduce risk of BPD and death in a multi-unit Australian survey [15]. In the above retrospective study, the decision to take antiviral treatment was based on the clinical experience of the doctors, which led to selection bias.

This systematic review has some limitations. First, most of the included studies were conducted in the USA, Europe, and Australia. Therefore, the results of this review should be generalized with caution when considering differences in healthcare settings. Second, the small number of studies with small sample sizes and high heterogeneity in diagnosing BPD on postnatal day 28 may affect the reliability and interpretability of the results. The included studies may have potential publication bias. Most of the studies were not adjusted for confounders, which may produce low-quality evidence when pooled. In addition, false-negative controls may have been present in the retrospective cohort, affecting the accuracy of the results.

Despite these limitations, we are the first review to systematically examine the association of viral infections with BPD. The studies we included were cohort designs, nearly half of which were prospective and highly similar in terms of population recruitment, exposure assessment, and outcome definitions. Nearly 90% of the studies in our meta-analysis were of good quality (NOS score 7–9). Heterogeneity was relatively low among studies with a BPD diagnosis at 36 weeks postmenstrual period.

## Conclusion

Summary evidence from observational studies suggests that viral infection is associated with an increased risk of BPD diagnosed at 36 weeks postmenstrual age (very low certainty of evidence, 13 studies, 6768 participants). The risk of BPD diagnosed at 36 weeks postmenstrual age may be more significant in preterm infants exposed to CMV. Clinicians should suspect that viral infections, particularly CMV infection, may be a trigger for BPD.

The results of our meta-analysis need to be validated in methodologically sound prospective studies with large samples. In the meantime, high-quality randomized controlled studies are conducted to investigate whether prevention or treatment of viral infections may reduce the risk of BPD. To further understand the causal relationship between viral infections and BPD, we can also try to establish experimental humanized animal models of BPD and lung organoid models to identify the molecular regulatory mechanisms by which viruses cause BPD. The development of microbiomics of the respiratory tract will also help to discover more links between viruses and BPD in preterm infants.

### Supplementary Information

Below is the link to the electronic supplementary material.

Supplementary file1 (DOCX 412 kb)

## Data Availability

No datasets were generated or analyzed during the current study.

## Abbreviations

BPD: Bronchopulmonary dysplasia
BW: Birth weight
CMV: Cytomegalovirus
ELISA: Enzyme-linked immunosorbent assay
GA: Gestational age
MOOSE: Meta-analysis of observational studies in epidemiology
NDI: Neurodevelopmental impairment
NOS: Newcastle-Ottawa Scale
OR: Odds ratio
PCR: Polymerase chain reaction
PRISMA: Preferred reporting items for systematic reviews and meta-analysis
VLBW: Very low birth weight
VPI: Very preterm infants

## References

[1] Gilfillan M, Bhandari A, Bhandari V (2021). Diagnosis and management of bronchopulmonary dysplasia. BMJ.

[2] Chen D, Chen J, Cui N, Cui M, Chen X, Zhu X, Zhu X (2020). Respiratory morbidity and lung function analysis during the first 36 months of life in infants with bronchopulmonary dysplasia (BPD). Front Pediatr.

[3] Malloy KW, Austin ED (2021). Pulmonary hypertension in the child with bronchopulmonary dysplasia. Pediatr Pulmonol.

[4] Li W, Wang Y, Song J, Zhang C, Xu Y, Xu F, Wang X, Zhu C (2023). Association between bronchopulmonary dysplasia and death or neurodevelopmental impairment at 3 years in preterm infants without severe brain injury. Front Neurol.

[5] Decollogne L, Epiard C, Chevallier M, Ego A, Alin L, Debillon T (2020). Neurodevelopmental impairment at 2 years of age in children born before 29 weeks’ gestation with bronchopulmonary dysplasia. Arch Pediatr.

[6] van Katwyk S, Augustine S, Thébaud B, Thavorn K (2020). Lifetime patient outcomes and healthcare utilization for bronchopulmonary dysplasia (BPD) and extreme preterm infants: a microsimulation study. BMC Pediatr.

[7] Salimi U, Dummula K, Tucker MH, Dela Cruz CS, Sampath V (2022). Postnatal sepsis and bronchopulmonary dysplasia in premature infants: mechanistic insights into "new BPD". Am J Respir Cell Mol Biol.

[8] Stolz C, Costa-Nobre DT, Sanudo A, de Lima Mota Ferreira DM, Sales Alves JM, dos Santos JP, Harumi Miyoshi M, de Mello Silva NM, de Godoi Melo FP, da Silva RVC, Barcala D, Vale MS, de Souza Rugolo LMS, Diniz EMA, Ribeiro M, Marba STM, Cwajg S, Duarte JLMB, Gonçalves Ferri WA, Procianoy RS, Anchieta LM, de Andrade Lopes JM, de Almeida MF, Guinsburg R (2023). Bronchopulmonary dysplasia: temporal trend from 2010 to 2019 in the Brazilian Network on Neonatal Research. Fetal Neonatal.

[9] Gobec K, Mukenauer R, Keše D, Erčulj V, Grosek Š, Perme T (2023). Association between colonization of the respiratory tract with Ureaplasma species and bronchopulmonary dysplasia in newborns with extremely low gestational age: a retrospective study.

[10] Imanishi Y, Hirata K, Nozaki M, Mochizuki N, Hirano S, Wada K (2021). The association between early gram-negative bacteria in tracheal aspirate cultures and severe bronchopulmonary dysplasia among extremely preterm infants requiring prolonged ventilation. Am J Perinatol.

[11] Xu Q, Yu J, Liu D, Tan Q, He Y (2022). The airway microbiome and metabolome in preterm infants: potential biomarkers of bronchopulmonary dysplasia. Front Pediatr.

[12] Bennett NJ, Tabarani CM, Bartholoma NM, Wang DL, Huang DN, Riddell SW, Kiska DL, Hingre R, Rosenberg HF, Domachowske JB (2012). Unrecognized viral respiratory tract infections in premature infants during their birth hospitalization: a prospective surveillance study in two neonatal intensive care units. J Pediatr.

[13] Sánchez Garciá L, Calvo C, Casas I, Pozo F, Pellicer A (2020). Viral respiratory infections in very low birthweight infants at neonatal intensive care unit: prospective observational study. BMJ Paediatr Open.

[14] Couroucli XI, Welty SE, Ramsay PL, Wearden ME, Fuentes-Garcia FJ, Ni JY, Jacobs TN, Towbin JA, Bowles NE (2000). Detection of microorganisms in the tracheal aspirates of preterm infants by polymerase chain reaction: association of adenovirus infection with bronchopulmonary dysplasia. Pediatr Res.

[15] Tapawan SJC, Bajuk B, Oei JL, Palasanthiran P (2023). Symptomatic postnatal cytomegalovirus infection in less than 32-week preterm infants: 13-year retrospective multicenter case-control study. Neonatology.

[16] Weimer KED, Kelly MS, Permar SR, Clark RH, Greenberg RG (2020). Association of adverse hearing, growth, and discharge age outcomes with postnatal cytomegalovirus infection in infants with very low birth weight. JAMA Pediatr.

[17] Prösch S, Lienicke U, Priemer C, Flunker G, Seidel WF, Krüger DH, Wauer RR (2002). Human adenovirus and human cytomegalovirus infections in preterm newborns: no association with bronchopulmonary dysplasia. Pediatr Res.

[18] Turner KM, Lee HC, Boppana SB, Carlo WA, Randolph DA (2014). Incidence and impact of CMV infection in very low birth weight infants. Pediatrics.

[19] Minihan L, Lee Oei J, Bajuk B, Palasanthiran P (2022). Postnatal cytomegalovirus infection: is it important? A 10-year retrospective case-control study of characteristics and outcomes in very preterm and very low birth weight infants. Pediatr Infect Dis J.

[20] Hernandez-Alvarado N, Shanley R, Schleiss MR, Ericksen J, Wassenaar J, Webo L, Bodin K, Parsons K, Osterholm EA (2021). Clinical, virologic and immunologic correlates of breast milk acquired cytomegalovirus (CMV) infections in very low birth weight (VLBW) infants in a newborn intensive care unit (NICU) setting. Viruses.

[21] Page MJ, McKenzie JE, Bossuyt PM, Boutron I, Hoffmann TC, Mulrow CD, Shamseer L, Tetzlaff JM, Akl EA, Brennan SE, Chou R, Glanville J, Grimshaw JM, Hróbjartsson A, Lalu MM, Li T, Loder EW, Mayo-Wilson E, McDonald S, McGuinness LA, Stewart LA, Thomas J, Tricco AC, Welch VA, Whiting P, Moher D (2021). The PRISMA 2020 statement: an updated guideline for reporting systematic reviews. BMJ.

[22] Brooke BS, Schwartz TA, Pawlik TM (2021). MOOSE reporting guidelines for meta-analyses of observational studies. JAMA Surg.

[23] Jobe AH, Bancalari E (2001). Bronchopulmonary dysplasia. Am J Respir Crit Care Med.

[24] Higgins RD, Jobe AH, Koso-Thomas M, Bancalari E, Viscardi RM, Hartert TV, Ryan RM, Kallapur SG, Steinhorn RH, Konduri GG, Davis SD, Thebaud B, Clyman RI, Collaco JM, Martin CR, Woods JC, Finer NN, Raju TNK (2018). Bronchopulmonary dysplasia: executive summary of a workshop. J Pediatr.

[25] Jensen EA, Dysart K, Gantz MG, McDonald S, Bamat NA, Keszler M, Kirpalani H, Laughon MM, Poindexter BB, Duncan AF, Yoder BA, Eichenwald EC, DeMauro SB (2019). The diagnosis of bronchopulmonary dysplasia in very preterm infants. An evidence-based approach. Am J Respir Crit Care Med.

[26] Inagaki K, Blackshear C, Hobbs CV (2019). Bronchopulmonary dysplasia in very preterm infants with symptomatic congenital cytomegalovirus infection: a propensity score-matched analysis. J Pediatr.

[27] Sawyer MH, Edwards DK, Spector SA (1987). Cytomegalovirus infection and bronchopulmonary dysplasia in premature infants. Am J Dis Child.

[28] Bimboese P, Kadambari S, Tabrizi SN, Garland SM, Tigg A, Lau R, Morley CJ, Curtis N (2022). Postnatal cytomegalovirus infection of Preterm and very-low-birth-weight infants through maternal breast milk: does it matter?. Pediatr Infect Dis J.

[29] Capretti MG, Lanari M, Lazzarotto T, Gabrielli L, Pignatelli S, Corvaglia L, Tridapalli E, Faldella G (2009). Very low birth weight infants born to cytomegalovirus-seropositive mothers fed with their mother’s milk: a prospective study. J Pediatr.

[30] Humberg A, Leienbach V, Fortmann MI, Rausch TK, Buxmann H, Müller A, Herting E, Härtel C, Göpel W (2018). Prevalence of congenital CMV infection and antiviral therapy in very-low-birth-weight infants: observations of the German neonatal network. Klin Padiatr.

[31] Meier J, Lienicke U, Tschirch E, Krüger DH, Wauer RR, Prösch S (2005). Human cytomegalovirus reactivation during lactation and mother-to-child transmission in preterm infants. J Clin Microbiol.

[32] Mukhopadhyay S, Meyer SA, Permar SR, Puopolo KM (2016). Symptomatic postnatal cytomegalovirus testing among very low-birth-weight infants: indications and outcomes. Am J Perinatol.

[33] Neuberger P, Hamprecht KA, Vochem M, Maschmann J, Speer CP, Jahn G, Poets CF, Goelz R (2006). Case-control study of symptoms and neonatal outcome of human milk-transmitted cytomegalovirus infection in premature infants. J Pediatr.

[34] Kelly MS, Benjamin DK, Puopolo KM, Laughon MM, Clark RH, Mukhopadhyay S, Brian Smith P, Permar SR (2015). Postnatal cytomegalovirus infection and the risk for bronchopulmonary dysplasia. JAMA Pediatr.

[35] Mani S, Hazra S, Hagan J, Sisson A, Nair J, Pammi M (2023). Viral infections and neonatal necrotizing enterocolitis: a meta-analysis. Pediatrics.

[36] Stark A, Cantrell S, Greenberg RG, Permar SR, Weimer KED (2021). Long-term outcomes after postnatal cytomegalovirus infection in low birthweight preterm infants: a systematic review. Pediatr Infect Dis J.

[37] Chaw PS, Hua L, Cunningham S, Campbell H, Mikolajczyk R, Nair H (2019). Respiratory syncytial virus-associated acute lower respiratory infections in children with bronchopulmonary dysplasia: systematic review and meta-analysis. J Infect Dis.

[38] Arroyo R, Kingma PS (2021). Surfactant protein D and bronchopulmonary dysplasia: a new way to approach an old problem. Respir Res.

[39] Letsiou E, Htwe YM, Dudek SM (2021). Secretory phospholipase A2 enzymes in acute lung injury. Cell Biochem Biophys.

[40] De Luca D, Lopez-Rodriguez E, Minucci A, Vendittelli F, Gentile L, Stival E, Conti G, Piastra M, Antonelli M, Echaide M, Perez-Gil J, Capoluongo ED (2013). Clinical and biological role of secretory phospholipase A2 in acute respiratory distress syndrome infants. Crit Care.

[41] Guaman MC, Pishevar N, Abman SH, Keszler M, Truog WE, Panitch H, Nelin LD (2021). Invasive mechanical ventilation at 36 weeks post-menstrual age, adverse outcomes with a comparison of recent definitions of bronchopulmonary dysplasia. J Perinatol.

[42] Isayama T, Lee SK, Yang J, Lee D, Daspal S, Dunn M, Shah PS (2017). Revisiting the definition of bronchopulmonary dysplasia. JAMA Pediatr.

[43] Jeon GW, Oh M, Chang YS (2021). Definitions of bronchopulmonary dysplasia and long-term outcomes of extremely preterm infants in Korean Neonatal Network. Sci Rep.

[44] Maltezou P-G, Kourlaba G, Kourkouni Ε, Luck S, Blázquez-Gamero D, Ville Y, Lilleri D, Dimopoulou D, Karalexi M, Papaevangelou V (2020). Maternal type of CMV infection and sequelae in infants with congenital CMV: systematic review and meta-analysis. J Clin Virol.

[45] Jones CE, Bailey H, Bamford A, Calvert A, Dorey RB, Drysdale SB, Khalil A, Heath PT, Lyall H, Ralph KMI, Sapuan S, Vandrevala T, Walter S, Whittaker E, Wood S (2023). Managing challenges in congenital CMV: current thinking. Arch Dis Child.

[46] Fourgeaud J, Boithias C, Walter-Nicolet E, Kermorvant E, Couderc S, Parat S, Pol C, Mousset C, Bussières L, Guilleminot T, Ville Y, Nkam L, Grimaldi L, Parodi M, Leruez-Ville M (2022). Performance of targeted congenital cytomegalovirus screening in newborns failing universal hearing screening: a multicenter study. Pediatr Infect Dis J.

[47] Josephson CD, Caliendo AM, Easley KA, Knezevic A, Shenvi N, Hinkes MT, Patel RM, Hillyer CD, Roback JD (2014). Blood transfusion and breast milk transmission of cytomegalovirus in very low-birth-weight infants. JAMA Pediatr.

[48] Hu X, Hu W, Sun X, Chen L, Luo X (2021). Transmission of cytomegalovirus via breast milk in low birth weight and premature infants: a systematic review and meta-analysis. BMC Pediatr.

[49] Ogawa R, Kasai A, Hiroma T, Tozuka M, Inaba Y, Nakamura T (2023). Prospective cohort study for postnatal cytomegalovirus infection in preterm infants. J Obstet Gynaecol Res.

[50] Huang T, Cai W, Ni C, Lai S, Lin S, Wang Q (2022). Changes in cytomegalovirus load in the breast milk of very/extremely premature infants and the effect of pasteurization and freeze–thawing on reducing viral load. Front Pediatr.

[51] Bapistella S, Hamprecht K, Thomas W, Speer CP, Dietz K, Maschmann J, Poets CF, Goelz R (2019). Short-term pasteurization of breast milk to prevent postnatal cytomegalovirus transmission in very preterm infants. Clin Infect Dis.

[52] Parker MG, Stellwagen LM, Noble L, Kim JH, Poindexter BB, Puopolo KM (2021). Promoting human milk and breastfeeding for the very low birth weight infant. Pediatrics.

[53] Lund A-M, Löfqvist C, Pivodic A, Lundgren P, Hård A-L, Hellström A, Hansen-Pupp I (2019). Unpasteurised maternal breast milk is positively associated with growth outcomes in extremely preterm infants. Acta Paediatr.

[54] Binte Abu Bakar SY, Salim M, Clulow AJ, Nicholas KR, Boyd BJ (2021). Human milk composition and the effects of pasteurisation on the activity of its components. Trends Food Sci Technol.

[55] Kilby MD, Ville Y, Acharya G (2019). Screening for cytomegalovirus infection in pregnancy. BMJ.

[56] Garofoli F, Civardi E, Zanette S, Angelini M, Perotti G, Zecca M, Lombardi G (2021). Literature review and an Italian hospital experience about post-natal CMV infection acquired by breast-feeding in very low and/or extremely low birth weight infants. Nutrients.

[57] Osterholm EA, Schleiss MR (2020). Impact of breast milk-acquired cytomegalovirus infection in premature infants: pathogenesis, prevention, and clinical consequences?. Rev Med Virol.

[58] Razonable RR, Humar A (2019). Cytomegalovirus in solid organ transplant recipients-guidelines of the American Society of Transplantation Infectious Diseases Community of Practice. Clin Transpl.

[59] Gantt S, Goldfarb DM, Park A, Rawlinson W, Boppana SB, Lazzarotto T, Mertz LM (2020). Performance of the Alethia CMV assay for detection of cytomegalovirus by use of neonatal saliva swabs. J Clin Microbiol.

[60] Dorfman L, Amir J, Attias J, Bilavsky E (2020). Treatment of congenital cytomegalovirus beyond the neonatal period: an observational study. Eur J Pediatr.

