# Supplementary material for: Association between viral infection and bronchopulmonary dysplasia in preterm infants: a systematic review and meta-analysis
Source: Eur J Pediatr. 2024 Apr 18;183(7):2965–81. doi: 10.1007/s00431-024-05565-9 (PMC11192663; doi:10.1007/s00431-024-05565-9)
Supplement: Supplementary file 1 — Supplementary file1 (DOCX 412 kb) [file 431_2024_5565_MOESM1_ESM.docx]

**Supplemental Information**

**Catalogue of supplementary information**

1. Appendix 1 - Search strategy for databases

2. Appendix 2 - Data items extracted from studies

3. Supplemental Table 1 - Studies excluded from the review

4. Supplemental Table 2 - Risk of bias assessment by Newcastle Ottawa Scale

5. Supplemental Figure 1 - Characteristics of included studies

6. Supplemental Figure 2 - Assessment of publication bias

7. Supplemental Table 3 - PRISMA 2020 Main Checklist

8. Supplemental Table 4 - PRISMA 2020 Abstract Checklist

**Appendix 1 - Search strategy for four databases**

| PubMed | | |
| --- | --- | --- |
| Search number | Query | Results |
| 11 | (((("Viruses"[MeSH Terms]) OR ("Virus Diseases"[MeSH Terms])) OR ("viral*"[Title/Abstract])) OR ("virus*"[Title/Abstract])) AND ((((("Bronchopulmonary Dysplasia"[MeSH Terms]) OR ("Dysplasia, Bronchopulmonary"[Title/Abstract])) OR ("bronchopulmonary dysplasia"[Title/Abstract])) OR ("chronic neonatal lung disease"[Title/Abstract])) OR ((((((((((neonat*[Title/Abstract]) OR (newborn*[Title/Abstract])) OR (preterm[Title/Abstract])) OR (prematur*[Title/Abstract])) OR (infant*[Title/Abstract])) OR ("Infant, Newborn"[MeSH Terms])) OR ("Infant, Premature"[MeSH Terms])) OR ("Infant, Extremely Premature"[MeSH Terms])) OR ("Infant, Premature, Diseases"[MeSH Terms])) AND (((("chronic lung disease"[Title/Abstract]) OR (BPD[Title/Abstract])) OR (CNLD[Title/Abstract])) OR ((outcom*[Title/Abstract]) AND ((respirat*[Title/Abstract]) OR (pulm*[Title/Abstract])))))) | 2,908 |
| 10 | (((("Bronchopulmonary Dysplasia"[MeSH Terms]) OR ("Dysplasia, Bronchopulmonary"[Title/Abstract])) OR ("bronchopulmonary dysplasia"[Title/Abstract])) OR ("chronic neonatal lung disease"[Title/Abstract])) OR ((((((((((neonat*[Title/Abstract]) OR (newborn*[Title/Abstract])) OR (preterm[Title/Abstract])) OR (prematur*[Title/Abstract])) OR (infant*[Title/Abstract])) OR ("Infant, Newborn"[MeSH Terms])) OR ("Infant, Premature"[MeSH Terms])) OR ("Infant, Extremely Premature"[MeSH Terms])) OR ("Infant, Premature, Diseases"[MeSH Terms])) AND (((("chronic lung disease"[Title/Abstract]) OR (BPD[Title/Abstract])) OR (CNLD[Title/Abstract])) OR ((outcom*[Title/Abstract]) AND ((respirat*[Title/Abstract]) OR (pulm*[Title/Abstract]))))) | 30,481 |
| 9 | (((((((((neonat*[Title/Abstract]) OR (newborn*[Title/Abstract])) OR (preterm[Title/Abstract])) OR (prematur*[Title/Abstract])) OR (infant*[Title/Abstract])) OR ("Infant, Newborn"[MeSH Terms])) OR ("Infant, Premature"[MeSH Terms])) OR ("Infant, Extremely Premature"[MeSH Terms])) OR ("Infant, Premature, Diseases"[MeSH Terms])) AND (((("chronic lung disease"[Title/Abstract]) OR (BPD[Title/Abstract])) OR (CNLD[Title/Abstract])) OR ((outcom*[Title/Abstract]) AND ((respirat*[Title/Abstract]) OR (pulm*[Title/Abstract])))) | 25,993 |
| 8 | ((("chronic lung disease"[Title/Abstract]) OR (BPD[Title/Abstract])) OR (CNLD[Title/Abstract])) OR ((outcom*[Title/Abstract]) AND ((respirat*[Title/Abstract]) OR (pulm*[Title/Abstract]))) | 156,099 |
| 7 | (outcom*[Title/Abstract]) AND ((respirat*[Title/Abstract]) OR (pulm*[Title/Abstract])) | 138,234 |
| 6 | ((("Viruses"[MeSH Terms]) OR ("Virus Diseases"[MeSH Terms])) OR ("viral*"[Title/Abstract])) OR ("virus*"[Title/Abstract]) | 1,940,549 |
| 5 | ((("Bronchopulmonary Dysplasia"[MeSH Terms]) OR ("Dysplasia, Bronchopulmonary"[Title/Abstract])) OR ("bronchopulmonary dysplasia"[Title/Abstract])) OR ("chronic neonatal lung disease"[Title/Abstract]) | 10,504 |
| 4 | ((((((((neonat*[Title/Abstract]) OR (newborn*[Title/Abstract])) OR (preterm[Title/Abstract])) OR (prematur*[Title/Abstract])) OR (infant*[Title/Abstract])) OR ("Infant, Newborn"[MeSH Terms])) OR ("Infant, Premature"[MeSH Terms])) OR ("Infant, Extremely Premature"[MeSH Terms])) OR ("Infant, Premature, Diseases"[MeSH Terms]) | 1,266,319 |
| 3 | (("chronic lung disease"[Title/Abstract]) OR (BPD[Title/Abstract])) OR (CNLD[Title/Abstract]) | 20,076 |
| 2 | (respirat*[Title/Abstract]) OR (pulm*[Title/Abstract]) | 1,214,673 |
| 1 | outcom*[Title/Abstract] | 2,432,357 |

| Cochrane library | | |
| --- | --- | --- |
| ID | Search | Hits |
| #1 | (viral*):ti,ab,kw OR (virus*):ti,ab,kw (Word variations have been searched) | 52121 |
| #2 | ("bronchopulmonary dysplasia"):ti,ab,kw OR ("chronic neonatal lung disease"):ti,ab,kw OR ("Dysplasia, Bronchopulmonary"):ti,ab,kw (Word variations have been searched) | 1844 |
| #3 | (BPD):ti,ab,kw OR ("chronic lung disease"):ti,ab,kw OR (CNLD):ti,ab,kw (Word variations have been searched) | 3486 |
| #4 | (respirat*):ti,ab,kw OR (pulm*):ti,ab,kw (Word variations have been searched) | 138908 |
| #5 | (outcom*):ti,ab,kw (Word variations have been searched) | 787178 |
| #6 | (neonat*):ti,ab,kw OR (newborn*):ti,ab,kw OR (preterm):ti,ab,kw OR (prematur*):ti,ab,kw OR (infant*):ti,ab,kw (Word variations have been searched) | 103220 |
| #7 | MeSH descriptor: [Viruses] explode all trees | 14579 |
| #8 | MeSH descriptor: [Virus Diseases] explode all trees | 42145 |
| #9 | MeSH descriptor: [Bronchopulmonary Dysplasia] explode all trees | 683 |
| #10 | MeSH descriptor: [Infant, Newborn] explode all trees | 20843 |
| #11 | MeSH descriptor: [Infant, Premature] explode all trees | 4977 |
| #12 | MeSH descriptor: [Infant, Premature, Diseases] explode all trees | 4109 |
| #13 | MeSH descriptor: [Infant, Extremely Premature] explode all trees | 336 |
| #14 | #4 AND #5 | 60213 |
| #15 | #14 OR #3 | 62543 |
| #16 | #10 OR #11 OR #12 OR #13 OR #6 | 103232 |
| #17 | #15 AND #16 | 8797 |
| #18 | #2 OR #9 | 1844 |
| #19 | #17 OR #18 | 9379 |
| #20 | #1 OR #7 OR #8 | 71323 |
| #21 | #20 AND #19 | 838 |

| Embase | | |
| --- | --- | --- |
| No | Query Results | Results |
| #17 | #15 AND #16 | 5,412 |
| #16 | #8 OR #9 | 3,047,420 |
| #15 | #13 OR #14 | 47,355 |
| #14 | #6 OR #7 | 18,374 |
| #13 | #11 AND #12 | 37,770 |
| #12 | #4 OR #5 | 1,503,697 |
| #11 | #3 OR #10 | 258,744 |
| #10 | #1 AND #2 | 232,412 |
| #9 | viral*:ti,ab,kw OR virus*:ti,ab,kw | 1,304,036 |
| #8 | virus'/exp OR 'virus infection'/exp | 2,853,550 |
| #7 | dysplasia, bronchopulmonary':ti,ab,kw OR 'bronchopulmonary dysplasia':ti,ab,kw OR 'chronic neonatal lung disease':ti,ab,kw | 13,344 |
| #6 | lung dysplasia'/exp | 16,104 |
| #5 | newborn'/exp OR 'prematurity'/exp OR 'extremely premature birth'/exp | 757,552 |
| #4 | neonat*:ti,ab,kw OR newborn*:ti,ab,kw OR preterm:ti,ab,kw OR prematur*:ti,ab,kw OR infant*:ti,ab,kw | 1,218,597 |
| #3 | chronic lung disease':ti,ab,kw OR bpd:ti,ab,kw OR cnld:ti,ab,kw | 29,891 |
| #2 | respirat*:ti,ab,kw OR pulm*:ti,ab,kw | 1,683,966 |
| #1 | outcom*:ti,ab,kw | 3,593,319 |

| Web of Science Core Collection | | |
| --- | --- | --- |
| No | Search | Results |
| 1 | outcom* (TS) | 3213970 |
| 2 | respirat* (TS) OR pulm* (TS) | 1355308 |
| 3 | "chronic lung disease" (TS) OR BPD (TS) OR CNLD (TS) | 22754 |
| 4 | neonat* (TS) OR newborn* (TS) OR preterm (TS) OR prematur* (TS) OR infant* (TS) OR "Infant, Newborn" (TS) OR "Infant, Premature" (TS) OR "Infant, Extremely Premature" (TS) OR "Infant, Premature, Diseases" (TS) | 1057409 |
| 5 | "Bronchopulmonary Dysplasia" (TS) OR "Dysplasia, Bronchopulmonary" (TS) OR "chronic neonatal lung disease" (TS) | 13055 |
| 6 | "Viruses" (TS) OR "Virus Diseases" (TS) OR "viral*" (TS) OR "virus*" (TS) | 1303542 |
| 7 | #1 AND #2 | 165993 |
| 8 | #3 OR #7 | 185900 |
| 9 | #4 AND #8 | 27362 |
| 10 | #5 OR #9 | 33579 |
| 11 | #10 AND #6 | 2490 |

**Appendix 2 - Data items extracted from included studies**

Each reviewer collected the following data items from each study:

1. Year of publication

2. Publication type

3. Study period

4. Aim of the study

5. Study design

6. Population

7. Setting

8. Country

9. Inclusion criteria and exclusion criteria

10. Sample size

11. Exposure definition

12. Outcome definition

13. Statistical methods used for analysis

14. Gestational age

15. Sex

16. Birth weight

17. Number of BPD cases in the exposed and unexposed

18. Author’s Conclusions

| Supplemental Table 1 - Studies excluded from the review | | |
| --- | --- | --- |
| Study No. | **Study ID** | **Reason for exclusion** |
| 1 | Bravo-Queipo-de-Llano, 2023 | Simply comparing the situation of viral respiratory infections before and after the COVID-19 pandemic |
| 2 | Buxmann, 2009 | This only describes five cases of premature infants with acquired CMV infection not involving BPD |
| 3 | Chua, 2022 | Case series |
| 4 | Deshpande, 2006 | Commentary |
| 5 | Gentile, 2020 | The study population consisted of premature infants and term infants during infancy, and BPD was not the outcome |
| 6 | Greenough, 2005 | The study population consisted of infants with BPD, and the outcome was not BPD |
| 7 | Hogg, 2000 | Commentary |
| 8 | Jian, 2021 | None of the exposed factors exhibited viral infection |
| 9 | Kimberlin, 2015 | Editorial |
| 10 | Kumar, 2022 | The study does not encompass BPD |
| 11 | Kumar, 2021 | The study does not encompass BPD |
| 12 | Martin, 1997 | Separate incidences of BPD were not reported for the case and control groups |
| 13 | Minihan, 2022 | This article's data partially overlaps with the dataset presented in Tapawan (2023). However, the dataset in Tapawan (2023) is more extensive and encompasses a longer duration of the research period |
| 14 | Piedra, 1992 | Included term infants |
| 15 | Poole, 2019 | The viral infection occurred subsequent to the diagnosis of BPD |
| 16 | Pryhuber, 2015 | Review |
| 17 | Resch, 2006 | The purpose of this study is not to investigate the relationship between viruses and BPD |
| 18 | Rogozina, 2023 | Due to the absence of data on gestational age at birth, it is impossible to ascertain whether the study population consisted of preterm infants |
| 19 | Ronchi, 2018 | The viral infection occurred subsequent to the diagnosis of BPD |
| 20 | Rose, 2021 | The purpose of this study is not to investigate the relationship between viruses and BPD |
| 21 | Smets, 1997 | The purpose of this study is not to investigate the relationship between viruses and BPD |
| 22 | Thwaites, 2004 | The study does not encompass BPD |
| 23 | Verboon-Maciolek, 2005 | The study does not encompass BPD |
| 24 | Whitley, 1980 | The study does not encompass BPD |
| 25 | Whitley, 1983 | Pharmacological interventional study |
| 26 | Yeo, 2018 | The discussion topic pertains to whether BPD serves as a risk factor for RSV infection |
| 27 | Yuksel, 1992 | Case series |
| 28 | Yuksel, 1994 | The outcome was not BPD |
| 29 | Zimmermann, 2022 | Case series, and the outcome was not BPD |
| 30 | Zinna, 2016 | The outcome was not BPD |

| Supplemental Table 2 - Risk of bias assessment by Newcastle Ottawa Scale | | | | | |
| --- | --- | --- | --- | --- | --- |
| Study No. | **Study ID** | **Selection** | **Comparability** | **Exposure or Outcome** | **NOS Score** |
| 1 | Bennett (2012) | 4 | 1 | 3 | 8 |
| 2 | Bimboese (2022) | 4 | 0 | 2 | 6 |
| 3 | Capretti (2009) | 4 | 0 | 3 | 7 |
| 4 | Couroucli (2000) | 4 | 0 | 2 | 6 |
| 5 | Hernandez-Alvarado (2021) | 4 | 0 | 3 | 7 |
| 6 | Humberg (2018) | 4 | 1 | 3 | 8 |
| 7 | Inagaki (2019) | 4 | 2 | 2 | 8 |
| 8 | Kelly (2015) | 4 | 2 | 3 | 9 |
| 9 | Meier (2005) | 4 | 0 | 3 | 7 |
| 10 | Mukhopadhyay (2016) | 3 | 1 | 3 | 7 |
| 11 | Neuberger (2006) | 3 | 1 | 3 | 7 |
| 12 | Prösch (2002) | 4 | 0 | 3 | 7 |
| 13 | Sánchez Garciá (2020) | 4 | 1 | 3 | 8 |
| 14 | Sawyer (1987) | 4 | 1 | 2 | 7 |
| 15 | Tapawan (2023) | 3 | 1 | 3 | 7 |
| 16 | Turner (2014) | 3 | 1 | 3 | 7 |
| 17 | Weimer (2020) | 4 | 2 | 3 | 9 |


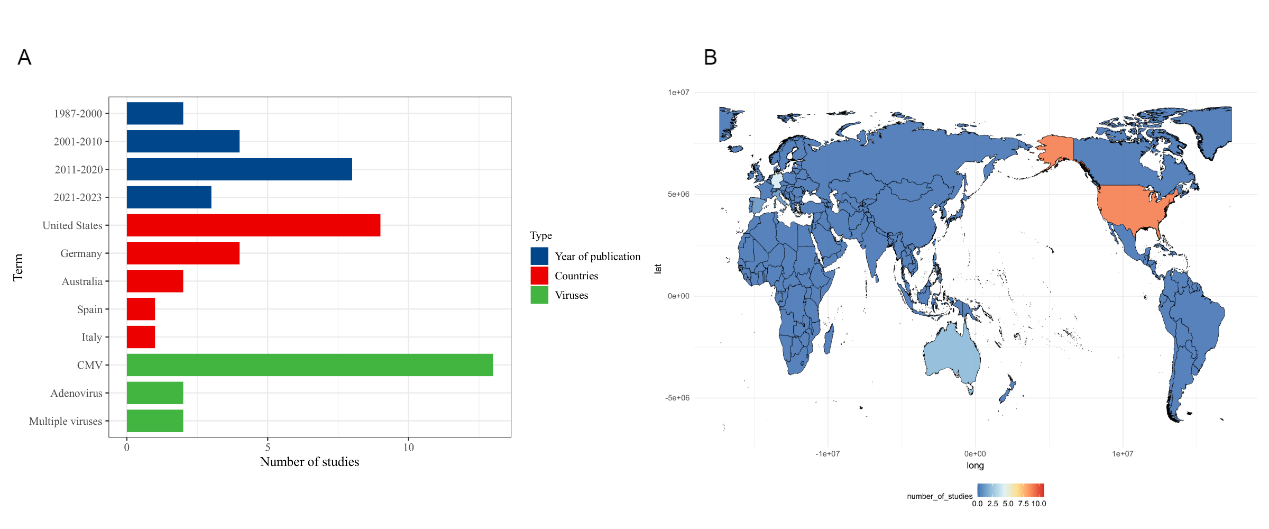


**Supplemental Figure 1** - Characteristics of included studies. (A) Bar chart with the x-axis indicating the number of included studies and the y-axis indicating entries for different publication years, countries and virus types. (B) Populated map indicating the distribution of countries in which the included studies were carried out, with the number of studies per country indicated by colour level.


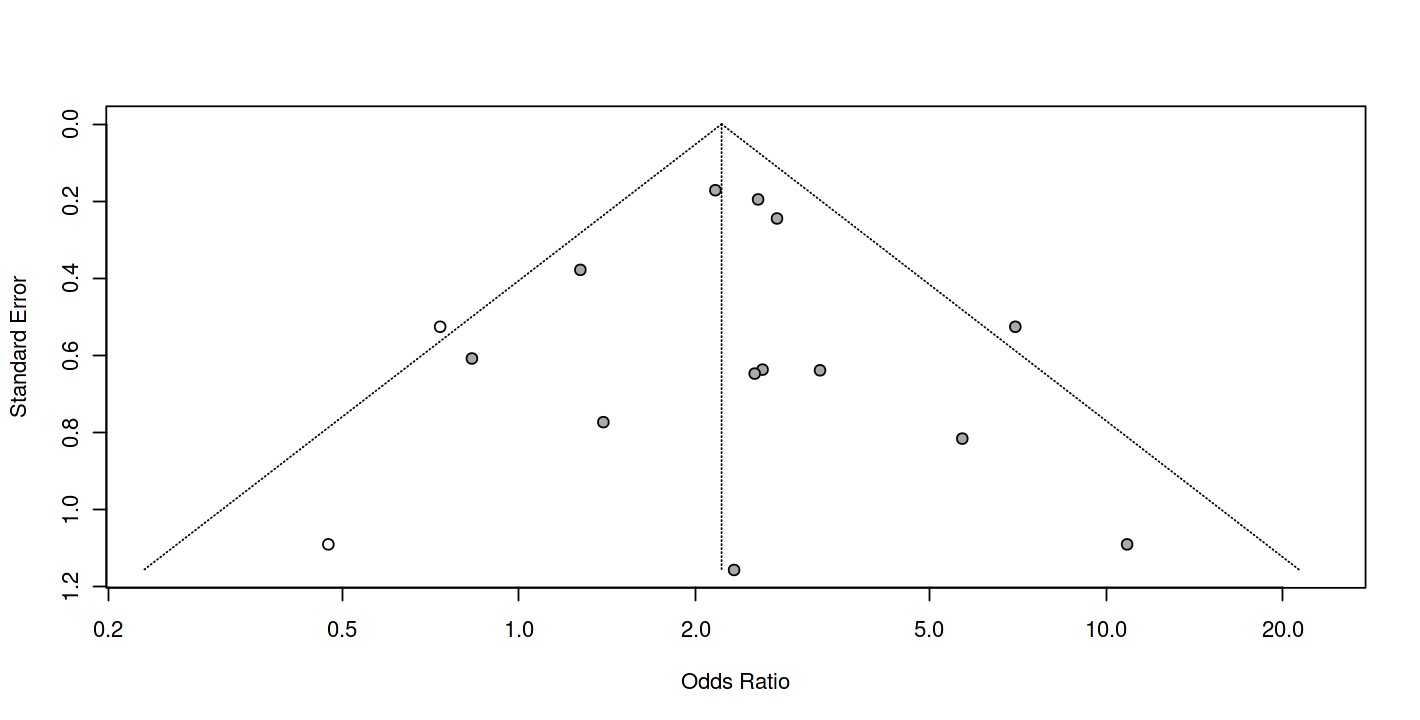


**Supplemental Figure 2** - Assessment of publication bias. The outcome in the 13 studies included in the meta-analysis was a diagnosis of BPD at 36 weeks postmenstrual age.


**Supplemental Table 3 - PRISMA 2020 Main Checklist**

| **Topic** | **No.** | **Item** | **Location where item is reported** |
| --- | --- | --- | --- |
| **TITLE** |  |  |  |
| **Title** | 1 | Identify the report as a systematic review. | 1 |
| **ABSTRACT** |  |  |  |
| **Abstract** | 2 | See the PRISMA 2020 for Abstracts checklist |  |
| **INTRODUCTION** |  |  |  |
| **Rationale** | 3 | Describe the rationale for the review in the context of existing knowledge. | 3-4 |
| **Objectives** | 4 | Provide an explicit statement of the objective(s) or question(s) the review addresses. | 5 |
| **METHODS** |  |  |  |
| **Eligibility criteria** | 5 | Specify the inclusion and exclusion criteria for the review and how studies were grouped for the syntheses. | 5 |
| **Information sources** | 6 | Specify all databases, registers, websites, organisations, reference lists and other sources searched or consulted to identify studies. Specify the date when each source was last searched or consulted. | 6 |
| **Search strategy** | 7 | Present the full search strategies for all databases, registers and websites, including any filters and limits used. | 6-7 |
| **Selection process** | 8 | Specify the methods used to decide whether a study met the inclusion criteria of the review, including how many reviewers screened each record and each report retrieved, whether they worked independently, and if applicable, details of automation tools used in the process. | 7 |
| **Data collection process** | 9 | Specify the methods used to collect data from reports, including how many reviewers collected data from each report, whether they worked independently, any processes for obtaining or confirming data from study investigators, and if applicable, details of automation tools used in the process. | 7 |
| **Data items** | 10a | List and define all outcomes for which data were sought. Specify whether all results that were compatible with each outcome domain in each study were sought (e.g. for all measures, time points, analyses), and if not, the methods used to decide which results to collect. | 6 |
|  | 10b | List and define all other variables for which data were sought (e.g. participant and intervention characteristics, funding sources). Describe any assumptions made about any missing or unclear information. | 6 |
| **Study risk of bias assessment** | 11 | Specify the methods used to assess risk of bias in the included studies, including details of the tool(s) used, how many reviewers assessed each study and whether they worked independently, and if applicable, details of automation tools used in the process. | 7 |
| **Effect measures** | 12 | Specify for each outcome the effect measure(s) (e.g. risk ratio, mean difference) used in the synthesis or presentation of results. | 7 |
| **Synthesis methods** | 13a | Describe the processes used to decide which studies were eligible for each synthesis (e.g. tabulating the study intervention characteristics and comparing against the planned groups for each synthesis (item 5)). | 7-8 |
|  | 13b | Describe any methods required to prepare the data for presentation or synthesis, such as handling of missing summary statistics, or data conversions. | 7-8 |
|  | 13c | Describe any methods used to tabulate or visually display results of individual studies and syntheses. | 7-8 |
|  | 13d | Describe any methods used to synthesize results and provide a rationale for the choice(s). If meta-analysis was performed, describe the model(s), method(s) to identify the presence and extent of statistical heterogeneity, and software package(s) used. | 7-8 |
|  | 13e | Describe any methods used to explore possible causes of heterogeneity among study results (e.g. subgroup analysis, meta-regression). | 7-8 |
|  | 13f | Describe any sensitivity analyses conducted to assess robustness of the synthesized results. | - |
| **Reporting bias assessment** | 14 | Describe any methods used to assess risk of bias due to missing results in a synthesis (arising from reporting biases). | 8 |
| **Certainty assessment** | 15 | Describe any methods used to assess certainty (or confidence) in the body of evidence for an outcome. | 11-12 |
| **RESULTS** |  |  |  |
| **Study selection** | 16a | Describe the results of the search and selection process, from the number of records identified in the search to the number of studies included in the review, ideally using a flow diagram. | 8 |
|  | 16b | Cite studies that might appear to meet the inclusion criteria, but which were excluded, and explain why they were excluded. | 8 |
| **Study characteristics** | 17 | Cite each included study and present its characteristics. | 8 |
| **Risk of bias in studies** | 18 | Present assessments of risk of bias for each included study. | 9-10 |
| **Results of individual studies** | 19 | For all outcomes, present, for each study: (a) summary statistics for each group (where appropriate) and (b) an effect estimate and its precision (e.g. confidence/credible interval), ideally using structured tables or plots. | 8-9 |
| **Results of syntheses** | 20a | For each synthesis, briefly summarise the characteristics and risk of bias among contributing studies. | 9-10 |
|  | 20b | Present results of all statistical syntheses conducted. If meta-analysis was done, present for each the summary estimate and its precision (e.g. confidence/credible interval) and measures of statistical heterogeneity. If comparing groups, describe the direction of the effect. | 10-11 |
|  | 20c | Present results of all investigations of possible causes of heterogeneity among study results. | 10-11 |
|  | 20d | Present results of all sensitivity analyses conducted to assess the robustness of the synthesized results. | - |
| **Reporting biases** | 21 | Present assessments of risk of bias due to missing results (arising from reporting biases) for each synthesis assessed. | 10 |
| **Certainty of evidence** | 22 | Present assessments of certainty (or confidence) in the body of evidence for each outcome assessed. | 11 |
| **DISCUSSION** |  |  |  |
| **Discussion** | 23a | Provide a general interpretation of the results in the context of other evidence. | 12-16 |
|  | 23b | Discuss any limitations of the evidence included in the review. | 16-17 |
|  | 23c | Discuss any limitations of the review processes used. | 16-17 |
|  | 23d | Discuss implications of the results for practice, policy, and future research. | 17-18 |
| **OTHER INFORMATION** |  |  |  |
| **Registration and protocol** | 24a | Provide registration information for the review, including register name and registration number, or state that the review was not registered. | 5 |
|  | 24b | Indicate where the review protocol can be accessed, or state that a protocol was not prepared. | 5 |
|  | 24c | Describe and explain any amendments to information provided at registration or in the protocol. | 5 |
| **Support** | 25 | Describe sources of financial or non-financial support for the review, and the role of the funders or sponsors in the review. | 23 |
| **Competing interests** | 26 | Declare any competing interests of review authors. | 23 |
| **Availability of data, code and other materials** | 27 | Report which of the following are publicly available and where they can be found: template data collection forms; data extracted from included studies; data used for all analyses; analytic code; any other materials used in the review. | 23 |

**Supplemental Table 4 - PRISMA 2020 Abstract Checklist**

| **Topic** | **No.** | **Item** | **Reported?** |
| --- | --- | --- | --- |
| **TITLE** |  |  |  |
| **Title** | 1 | Identify the report as a systematic review. | Yes |
| **BACKGROUND** |  |  |  |
| **Objectives** | 2 | Provide an explicit statement of the main objective(s) or question(s) the review addresses. | Yes |
| **METHODS** |  |  |  |
| **Eligibility criteria** | 3 | Specify the inclusion and exclusion criteria for the review. | Yes |
| **Information sources** | 4 | Specify the information sources (e.g. databases, registers) used to identify studies and the date when each was last searched. | Yes |
| **Risk of bias** | 5 | Specify the methods used to assess risk of bias in the included studies. | Yes |
| **Synthesis of results** | 6 | Specify the methods used to present and synthesize results. | Yes |
| **RESULTS** |  |  |  |
| **Included studies** | 7 | Give the total number of included studies and participants and summarise relevant characteristics of studies. | Yes |
| **Synthesis of results** | 8 | Present results for main outcomes, preferably indicating the number of included studies and participants for each. If meta-analysis was done, report the summary estimate and confidence/credible interval. If comparing groups, indicate the direction of the effect (i.e. which group is favoured). | Yes |
| **DISCUSSION** |  |  |  |
| **Limitations of evidence** | 9 | Provide a brief summary of the limitations of the evidence included in the review (e.g. study risk of bias, inconsistency and imprecision). | Yes |
| **Interpretation** | 10 | Provide a general interpretation of the results and important implications. | Yes |
| **OTHER** |  |  |  |
| **Funding** | 11 | Specify the primary source of funding for the review. | No |
| **Registration** | 12 | Provide the register name and registration number. | No |
